# Historical Synthesis-Analysis of Changes in Grain Nitrogen Dynamics in Sorghum

**DOI:** 10.3389/fpls.2016.00275

**Published:** 2016-03-08

**Authors:** Ignacio A. Ciampitti, P. V. Vara Prasad

**Affiliations:** Department of Agronomy, Kansas State University, ManhattanKS, USA

**Keywords:** nitrogen use efficiency, sorghum bicolor, grain yield, nitrogen uptake, grain nitrogen, nutrient partitioning

## Abstract

Unraveling the complexity underpinning nitrogen (N) use efficiency (NUE) can be physiologically approached via examining grain N sources and N internal efficiency (NIE) (yield to plant N content ratio). The main objective of this original research paper is to document and understand sorghum NUE and physiological mechanisms related to grain N dynamics. The study of different grain N sources, herein defined as the reproductive-stage shoot N remobilization (Remobilized N), reproductive-stage whole-plant N content (Reproductive N), and vegetative-stage whole-plant N content (Vegetative N), was pursued with the goal of synthesizing scientific literature for sorghum [*Sorghum bicolor* (L.) Moench] crop. A detailed literature review was performed and summarized on sorghum NUE (13 studies; >250 means) with three Eras, defined by the year of the study, named as Old Era (1965–1980); Transient Era (1981–2000); and New Era (2001–2014). The most remarkable outcomes from this synthesis were: (1) overall historical (1965–2014) cumulative yield gain was >0.5 Mg ha^-1^ (yields >7 Mg ha^-1^); (2) NIE did not change across the same time period; (3) grain N concentration (grain %N) accounted for a large proportion (63%) of the variation in NIE; (4) NIE increased as grain %N diminished, regardless of the Eras; (5) Remobilized N was strongly (>*R*^2^ 0.6) and positively associated with Vegetative N, presenting a unique slope across Eras; and (6) a trade-off was documented for the Remobilized N and Reproductive N (with large variation, <*R*^2^) relationship, suggesting complex regulation processes governing N forces. Improvements in NUE are subjected to the interplay between N supply (N from non-reproductive organs) and grain N demand, sink- (driven by grain number) and source-modulated (via restriction of grain N demand).

## Introduction

For the last six decades, US sorghum (*Sorghum bicolor* L. Moench) improvement has been related to targeted modifications in genotype (G component) and management practices (M component), such as (a) fertilization rates, (b) irrigation, and (c) tillage practices (Eghball and Power, 1995; Duvick, 1999; Assefa and Staggenborg, 2010). A long-term study conducted in Texas (1939–1997) documented yield improvements were mainly related to the introduction of new sorghum hybrids, water conditions at planting, better weed (herbicide) control and conservation practices such as zero tillage (Unger and Baumhardt, 1999). One-third (35–40%) of the overall yield improvement could be attributed to the G component and two-thirds to interaction between the M factor by the environment (E) component (Duvick, 1999; Unger and Baumhardt, 1999; Assefa and Staggenborg, 2010). Similar yield gains were also documented by Miller and Kebede (1984). From 1950 to 1999, Mason et al. (2008) documented a slower rate of yield improvement in sorghum relative to maize (*Zea mays* L.). Overall, sorghum yield improvement seemed to be primarily achieved by gains under environmental stress and low yielding environments rather than by modifications or improvements on maximum yield potential (Assefa and Staggenborg, 2010).

Sorghum is a C4 (Kranz leaf anatomy) erect plant constituted from a main stem, leaf canopy, head, and tiller organs. The final tiller number is dependent on the genotype, temperature, and nutrient resources. Final plant size as related to the aboveground portion is dependent on the plant response to photoperiod sensitivity and growing conditions; while the belowground section is composed by extensive fibrous root systems. For the main stem, leaf area increases until full expansion of the flag leaf occurs; a waxy bloom often covered leaf sheath and stem organs. Grains are developed in the head organ (in the uppermost section of the plant) after flowering time. The plant has several uses as for grain, forage, feed, and bioenergy. As related to the nutritional value, sorghum contains approximately 11–13% protein, which is free of gluten presenting an advantage as a food supply from people suffering from celiac problems. Sorghum presents a good nutritional value, with an overall grain composition of 70–80% carbohydrate, 2–5% fat, 1–3% fiber, and 1–2% ash.

Physiologically, sorghum presents a plasticity connected to the capacity of the plant to compensate and adjust its growth based on the resources available at the plant-scale (Heinrich et al., 1983). Superior water use efficiency for grain sorghum as relative to other crops (such as corn and soybean), expressed as higher yield per unit of water, was documented in low-yielding, and water-limited environments (Stone et al., 2006). Historically, sorghum genetic improvement is related to changes in aboveground biomass production (increased leaf to stem ratio and higher leaf mass), longer panicle length, decrease in peduncle length, and superior root mass (Assefa and Staggenborg, 2011). Sorghum yield improvement is tightly connected to changes in number of panicles per unit land area, increased kernel numbers, and increased final total grain weight. Production factors such as non-uniform stands, row spacing, plant population, weed-competition, defoliation, water availability, and N applications directly affect yield components (Stickler and Wearden, 1965; Rajewski et al., 1991; M’Khaitir and Vanderlip, 1992; Norwood, 1992; Larson and Vanderlip, 1994; Limon-Ortega et al., 1998) impacting yield potential of grain sorghum. The E factor (e.g., water and temperature) exerts a large influence; thus, endpoint sorghum productivity may be considered the outcome of a complex G × E × M interaction.

From a plant nutrition perspective, nitrogen (N) is the main nutrient influencing plant growth, aboveground biomass, and yield (Roy and Wright, 1973; Kamoshita et al., 1998; Borrell and Hammer, 2000; Wortmann et al., 2007; van Oosterom et al., 2010; Kaizzi et al., 2012; Mahama et al., 2014). For modern sorghum hybrids, N application improved yields via modification of aboveground biomass, seed number, and grain HI (Mahama et al., 2014). Improvement of NUE (yield to available N ratio) can be understood via dissecting NUE into two components: N recovery efficiency (NRE, plant N uptake to soil N supply) and (NIE, yield to plant N uptake ratio) (Ciampitti and Vyn, 2012). Historical NUE gains in maize were primarily explained by improvements in N uptake, and consequently, NIE (Ciampitti and Vyn, 2012, 2013, 2014). Nonetheless, yield improvement has been indirectly accompanied by decreases in grain %N (Duvick and Mickelson, 1997; Ciampitti and Vyn, 2012, 2013). Changes in NIE over time are not yet documented for sorghum. Therefore, detailed review and research on NUE, its components, and grain N pathways is critical and required. For maize, a trade-off was documented for the Remobilized N and Reproductive N (Ciampitti and Vyn, 2013). A scientific knowledge gap exists for better understanding of these processes for sorghum NUE and concomitant yield improvement.

The main objective of this original research paper is to document and understand sorghum NUE and physiological mechanisms related to grain N dynamics. Understanding historical changes of grain N sources (organ size and potential genotypic variation) and physiological strategies of the sorghum for securing this N demand will facilitate parallel improvements in NUE and grain yield.

## Materials and Methods

### Data Standardization

Information gathered from scientific literature over the past several decades was synthesized and presented in **Table 1**. A data inclusion criteria implemented in a previous synthesis analysis was followed (Ciampitti and Vyn, 2013) for this study. Briefly, information was included in the database if specific criteria were met; primarily focused on completeness of the information (e.g., data on grain yield, plant N content at flowering, plant N fractions = grain and stover N content at maturity). A total of 13 research studies that satisfied all requirements were selected for the analyses, comprising >250 treatment means (**Table 1**). Unpublished thesis reports, current on-going research (Ciampitti and Prasad, Unpublished) and research studies not yet published in scientific journals (three M.S. and three Ph.D. thesis dissertations) were also included to secure the “unbiasedness” and avoid a misinterpretation (when only significant outcomes are published) of overall population effects (McLeod and Weisz, 2004). Geographically, the studies are representing the main sorghum producing areas around the globe such as US Great Plains (Nebraska, Kansas, and Texas), Australia, and India. In relative terms, the database is composed with a frequency of observations (treatment means) of 62% Kansas, 15% Australia, 12% Nebraska, 6% Texas, and 5% India (**Table 1**). Due to lack of balance geographic number of observations, this factor was not analyzed individually, but included in the historical evaluation (without further separation). The main challenge in evaluating, selecting, and synthesizing information for sorghum NUE was the lack of information for reproductive and non-reproductive N content at varying phenological stages. For exploratory and statistical evaluation (database distribution and normality), the database was arbitrarily divided into three Eras, based on the study year, named as Old Era (1965–1980; *n* = 87 treatment means); Transient Era (1981–2000; *n* = 85); and New Era (2001–2014; *n* = 86). Miller and Kebede (1984) documented a similar genetic yield gain for sorghum from 1960 to 1980. Mason et al. (2008) documented a historical yield improvement from 1980 to 2000. In the last years (2001–2009), a consistent positive yield trend was recorded for main sorghum producing countries around the globe such as Australia, US, India, Brazil, Burkina Faso, Ethiopia, and Mexico (Rakshit et al., 2014), which is related to the interaction between genetic improvement and use of best management practices. For the US Great Plain region, hybrids accounted for only about 15% of the total sorghum seed planted in 1957. But by 1960, sorghum hybrids had rapid acceptance, accounting for 95% of the area (Maunder, 1998, 1999).

**Table 1:** Number of study, site/country, author, design, year of experimentation, number of genotypes, and characteristics for each different sorghum experiments.

No	Site/ Country	Author	Design	Year	No Genotypes	Main characteristics
1	Kansas, USA	Koch, 1966 (M.S. Thesis)	Split-plot	1964–1965	Six genotypes (three maturity groups)	Effect of maturity and plant density on N uptake and NUE
2	New Delhi, India	Roy and Wright, 1973	Complete factorial in randomized blocks	1967–1968	One genotype ‘CSH-I’	Nutrient uptake and partitioning
3	Kansas, USA	Stützel, 1981 (M.S. Thesis)	Alternated rows	1980	Three genotypes (early, medium and late)	Sorghum and millet intercropping: biomass, nutrient uptake, and yield
4	Nebraska, USA	Fernandez, 1987 (Ph.D. Dissertation)	Randomized complete block	1985	Four genotypes	Study of NUE and its physiological mechanisms
5	Texas, USA	Lavelle, 1987 (M.S. Thesis)	Randomized complete block	1983–1984	Four genotypes	NUE among sorghum genotypes (0 and 180 kg ha^-1^)
6	Nebraska, USA	Masi, 1997 (Ph.D. Dissertation)	Randomized complete block	1992–1993	10 genotypes (two hybrids)	Root systems and N uptake in diverse sorghum genotypes
7	Gatton, Australia	Kamoshita et al., 1998	Split-plot	1995–1996	Four genotypes (two early and two late maturing)	Hybrid × Nitrogen levels (0 and 240 kg ha^-1^) under low N
8	Nebraska, USA	Traore, 1998 (Ph.D. Dissertation)	Randomized complete block	1992–1993 1994–1996	15 genotypes (four hybrids)	Physiological contributions to NUE (genotypes)
9	Warwick, Australia	Borrell and Hammer, 2000	Split-plot	1994–1995	Nine hybrids	N and stay-green characterization
10	Gatton, Australia	van Oosterom et al., 2010	Split-plot	1999	Three genotypes (senescent, stay-green, and RUE^∗^)	Study of reproductive N: three hybrids × N rates (0, 44, and 353 kg ha^-1^)
11	Kansas, USA	Mahama et al., 2014	Split-plot in randomized complete block	2010–2011	12 genotypes (six hybrids)	Hybrid and inbreds, three fertilizer N rates (0, 45, and 90 kg ha^-1^)
12	Udaipur, India	Sumeriya et al., 2014	Split-plot	2004, 2005, 2006	One genotype	Soil moisture conservation practice
13	Kansas, USA	Ciampitti and Prasad, unpublished	Randomized complete block	2014	Four genotypes	Germplasm evaluation: NUE, N uptake and partitioning

*^∗^RUE, Indian hybrid with high radiation use efficiency (RUE).*

### Database Description: Parameters Evaluated

Biomass and plant N content was summarized from previously described studies (**Table 1**). For the purpose of this document, plant biomass refers to the mass accumulation in all aboveground plant fractions (e.g., stem, leaves, and head), excluding the below ground fraction of roots. Similarly, the plant N content considers all aboveground plant fractions and their related N content. Plant N content was calculated by multiplying the biomass by its respective N concentration (dry mass basis), if the plant N content was not explicitly provided in the particular research study. Grain and N harvest indices (HI) were calculated following the equations described by Ciampitti and Vyn (2013). Briefly, if grain HI and/or NHI was not directly reported, it was calculated as follows:

$$\begin{array}{c} Grain\;HI\;=\;Yield/Plant\;Biomass\;(stover:leaf+stem+grain)\\ NHI=\;Grain\;Ncontent/Plant\;Ncontent\;\;\;\;\; \end{array}$$

in which the Yield refers to the final grain yield at the end of the season (harvest maturity), adjusted by a constant moisture content (135 g kg^-1^ moisture content) and the plant biomass is expressed in dry weight basis. Both grain and plant N content are expressed in dry weight basis and determined at the end of the season.

Vegetative-stage aboveground biomass (Vegetative Biomass) and N content (Vegetative N) was obtained from all research studies involving biomass and N accumulation from emergence to flowering. Similarly, the reproductive-stage aboveground biomass (Reproductive Biomass) and N content (Reproductive N) involves biomass and N accumulation from flowering until the end of the season. The term named “Remobilized N” was calculated using the balance approach as follows (Ciampitti and Vyn, 2013):

Remobilized N=Vegetative N−Stover N,

in which vegetative N (all plant) at flowering and the stover N at maturity (leaf + stem).

As previously documented by Ciampitti and Vyn (2013), this so-called “balance” approach for estimating N remobilization is less accurate (e.g., sampling error) and more labor intensive (sampling at flowering) than the stable isotopic N method. The balance approach presents constrains related to potential issues related to sampling error due to proper identification of growth stages and temporal-data aggregation (Kichey et al., 2007). Nonetheless, this approach is legitimate and commonly utilized for estimating on-farm N remobilization.

The reproductive N was estimated as follows:

Reproductive N=Plant N−Vegetative N

in which the plant N (leaf + stem + head, including grain, fractions) at maturity.

Nitrogen internal efficiency was calculated as the yield to the plant N content ratio (Ciampitti and Vyn, 2012) all obtained at harvest maturity. Grain %N was calculated as the grain N content to yield ratio (if the information was not explicitly documented), both obtained at maturity.

$$\mathrm{NIE}(grain yield/plant N)= NHI(grain N/plant N)\;\times \;{\left[\mathrm{grain}\%N(grain N/grain yield)\right]}^{-1}.$$

### Descriptive Analysis: Database Summary

For all the data, a descriptive analysis was performed involving the total number of observations (and units), and calculation of mean, standard deviation (SD), minimum, 25–75% quartile, median, and maximum (**Table 2**). Box-plots was calculated for the yield factor across different historical Eras (GraphPad Prism 6; Motulsky and Christopoulos, 2003). For the entire database, histograms were developed using the “hist” function from the R program (R Development Core Team, 2009) to graphically show the grain yield and plant N content distribution. For model analyses (historical comparison between slopes), linear components were tested (*F*-test, Mead et al., 1993), and selected models were compared with a global fit (GraphPad Prism 6, Motulsky and Christopoulos, 2003). For the entire database, number of observations was not constant since not all parameters were reported or collected in all the studies (essential factors for the synthesis analyses). Grain yield and plant N content (**Figure 2A**) and NIE versus grain %N (**Figure 2B**) relationships were documented in per-unit area across historical Eras, with relative NHI or grain yield as data point size. For the associations Remobilized N versus Vegetative N, and Reproductive N versus Remobilized N (**Figures 3A,B**), the bubble graph technique was implemented (R program, R Development Core Team, 2009) to portray these relationships as a function of a third factor whereby different sizes refer to the grain yield (e.g., larger bubble sizes, high yields). Similar procedure was also implemented for the associations presented in **Figure 2**.

**Table 2:** Descriptive summary statistics of the synthesis (1965–2014) Eras relative to sorghum grain yield, Plant N, whole-plant biomass (Biomass) at different phenological stages (flowering and maturity, all on dry basis) and variables related to the partitioning components of yield and N and to the N use efficiency.

Parameter	Unit	*n*	Mean	*SD*	Minimum	25% Q	Median	75% Q	Maximum
Grain yield	Mg ha^-1^	258	5.2	2.4	1.2	3.4	4.9	6.8	20.9
Grain HI	Dimensionless	183	0.42	0.11	0.12	0.34	0.44	0.49	0.68
1000-Grain Weight	g 1000 seeds	154	24.9	3.5	2.6	23.3	25.1	27.2	32.3
Plant density	pl m^-2^	258	11.0	5.1	3.5	6.8	12.0	13.0	30.0
Vegetative BM	g m^-2^	220	511	362	34	112	541	791	2037
Plant BM	g m^-2^	183	1217	505	440	847	1150	1451	4444
Vegetative N	g m^-2^	258	9.4	5.0	2.4	5.5	8.7	12.1	26.0
Stover N	g m^-2^	258	5.0	2.9	0.5	2.6	4.3	7.0	13.5
Grain N	g m^-2^	258	8.8	4.8	1.9	5.0	7.1	13.0	33.6
Plant N	g m^-2^	258	13.8	6.9	3.6	7.8	12.8	18.7	43.7
NHI	Dimensionless	258	0.64	0.11	0.22	0.57	0.65	0.71	0.96
Remobilized N	g m^-2^	258	0.6	4.6	-13.7	-1.9	0.5	3.0	12.7
Reproductive N	g m^-2^	258	4.4	3.3	-5.8	2.1	3.5	6.6	18.3
NIE	g g^-1^	258	42	14	8	32	37	51	83
Grain %Nc	mg g^-^	256	16.5	4.9	5.2	12.5	16.9	19.4	37.0

*Grain HI, grain harvest index (yield to aboveground biomass –BM- ratio); Vegetative BM, whole-plant aboveground biomass at flowering time; Plant BM, whole-plant aboveground biomass at physiological maturity; Vegetative N, vegetative-stage whole-plant aboveground N content at flowering; Stover N, stover N content (Plant BM minus grain) at physiological maturity; Grain N, grain N content at physiological maturity; Plant N, whole-plant aboveground N content at physiological maturity; NHI (Grain N to Plant N ratio) determined at the end of the season; Remobilized N, reproductive-stage shoot N remobilization; Reproductive N, reproductive-stage whole-plant N content determined after flowering; NIE, Nitrogen Internal Efficiency, calculated as the yield to the Plant N ratio at physiological maturity; Grain %Nc, grain N concentration at physiological maturity.*

## Results

### Descriptive Analysis: Sorghum Database

**FIGURE 1:**
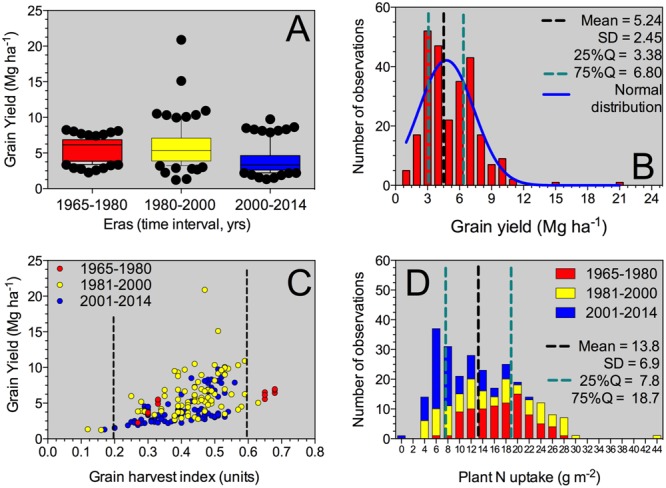
**Box-plot for grain yield factor across all Eras **(A)**, histogram for the grain yield parameter **(B)**, yield versus grain NHI relationship **(C)**, and histogram for the plant N uptake at maturity including all Eras evaluated in this synthesis-analysis.** For the box-plot **(A)**, the solid line indicates the mean value for grain yield. The box boundaries indicate the upper and lower quartiles, the whisker caps indicate 90th and 10th percentiles, and the circles indicate observations below and above those percentiles. For grain yield histogram **(B)**, mean, standard deviation (SD), 25–75% quartile and normal distribution (Gaussian fit) was performed. For grain yield versus grain HI **(C)**, information was individualized (identified by color) and separated by Eras (Old Era = 1965–1980; Transient Era = 1981–2000; New Era = 2001–2014). For plant N uptake histogram **(D)**, mean, SD, 25–75% quartile was determined for the entire database of plant N uptake, with Era separation.

Historical sorghum yield was 5.2 Mg ha^-1^ with a SD of 2.4 Mg ha^-1^ (25%Q = 3.4 Mg ha^-1^; 75%Q = 6.8 Mg ha^-1^) (**Table 2**). Maximum yield was close to 21 Mg ha^-1^; while minimum grain yield was of 1.2 Mg ha^-1^, representing a yield gap of 20 Mg ha^-1^. Historical yield changes are not necessarily reflecting changes in sorghum germplasm or management practices, but strictly representing the observations collected from this analysis. In the descriptive analysis, low yielding means (<3 Mg ha^-1^) for the Old Era (1965–1980 years; *n* = 87) represented only 6% of the category with an overall yield of 2.7 Mg ha^-1^; while for the New Era (2000–2014 years; *n* = 87), those data points represented 35%, averaging 2.4 Mg ha^-1^ (**Figure 1A**). On the opposite yielding range, for medium- to high-yielding treatment means (>7 Mg ha^-1^), the Old Era presented 18% of observations with an overall yield of 7.5 Mg ha^-1^; while the New Era has 14% within this category with a mean yield of 8.1 Mg ha^-1^.

Across all Eras, grain yield presented a normal distribution (mean = 4.8 Mg ha^-1^), with a tendency to high values (positively skewed distribution, skewness = 1.5) and more concentrated, 25% quartile, 25%Q = 3.4 Mg ha^-1^; 75%Q = 6.8 Mg ha^-1^ (leptokurtic, peaked distribution, kurtosis = 6.1) (**Figure 1B**). Yield was related (positive response) to the grain HI (grain yield to plant biomass ratio), with more variability in yield as grain HI increases above 0.30 (**Figure 1C**). Overall grain HI was 0.42 with a broad variation range (0.12 to 0.68 units; **Table 2**). Grain yields above 10 Mg ha^-1^ presented grain HI values ranging from 0.4 to 0.6 units (**Figure 1C**). The most recent two Eras (Transient and Modern Eras) did not portray a clear distinction for the grain yield versus grain HI relationship. The seed size, represented by the 1,000-grain weight, varied from 2.6 to 32.3 g with an average of 24.9 g (**Table 2**), reflecting a “flat” trend with the grain yield trait (data not shown). The head number factor was not tightly connected with the plant density, but instead was influenced by the tillering ability of each hybrid. Overall mean plant density observed was 11 plants m^-2^ broadly ranging (±5 plants m^-2^), with a distribution greatly concentrated (25–75Q) between 7 and 13 plants m^-2^ (**Table 2**).

### Plant N content and Grain Yield Relationship

Plant N content (aboveground whole-plant N at maturity) varied with the grain yield factor. Mean plant N content was 13.8 g m^-2^ (25 to 75%Q = 7.8 to 18.7 g m^-2^, respectively) with a distribution governed by the Era classification. For the Old Era (1965–1980 years), plant N content distribution was more flat (platykurtic, less peaked) and biased toward greater plant N content values typically related to greater yield (red columns, **Figure 1D**). On the opposite side, for the Modern Era (2001–2014 years), plant N content peaked toward lower values, more concentrated around 6 to 8 g m^-2^ (blue columns, **Figure 1D**). Overall plant biomass (aboveground whole-plant biomass at maturity, plant BM), presented a value of 1217 g m^-2^ (**Table 2**) with larger plant size connected to higher plant N content.

**FIGURE 2:**
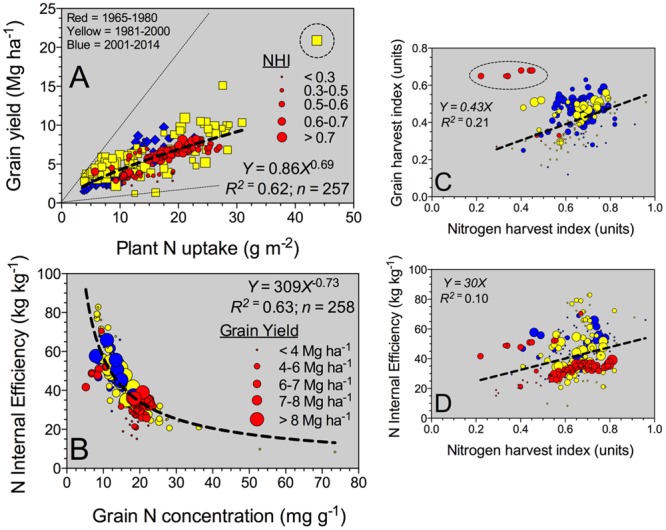
**Relationship between yield and plant N uptake **(A)** and nitrogen (N) internal efficiency (NIE, yield to plant N uptake ratio) versus grain %N **(B)**, grain HI (grain yield to plant biomass, BM) **(C)**, and NIE versus NHI (grain N to plant N content) **(D)** all determined at maturity, for all Eras (time interval from 1965 to 2014 years).** For **(A)**, bubble sizes refer to the different NHI values (larger bubble sizes for greater NHI). For panels **(B–D)**, bubble sizes refer to diverse sorghum grain yield values (larger bubble sizes for high-yielding values).

The grain yield to plant N content relationship (slope = NIE) fitted an exponential growth model (**Figure 2A**). In general, superior grain yield was achieved by increasing plant N content at maturity, regardless of the Eras. High-yielding environments (>8 Mg ha^-1^) presented a plant N content at or above 25 g m^-2^ (**Figure 2A**). Overall, NIE was 42 kg kg^-1^, but broadly ranging, 10-fold variation from min. to max. (8–83 kg kg^-1^) values (**Table 2**). Two components are part of the NIE, the NHI and the grain %N both determined at maturity. Superior NIE was negatively and strongly (*R*^2^ > 0.60; *n* = 258; *P* < 0.001) related to grain %N; thus, high N efficiency was achieved lowering grain %N at the same time (with yields ranging from 4 to 6 Mg ha^-1^; **Figure 2B**). A unique model was fit for the NIE versus grain %N (*Y* = β2^∗^*X*^β1^), with similar allometric coefficients (β1) for the last two Eras (1981–2000, 2001–2014). Historically, a large improvement in the NIE variation accounted by grain %N (*R*^2^ = 0.35 for 1965–1980 to *R*^2^ = 0.55 for 2001–2014) was documented. Additionally, biomass and N partitioning were weakly associated as reflected by the positive relationship between grain HI and NHI (**Figure 2C**). On the counterpart of the NIE term, NHI was positively related to grain HI, but larger variation was faced (**Figure 2C**). Critically, both NHI and grain %N were unrelated (data not shown, *R*^2^ = 0.01). In summary, grain %N was the main term accounted for most of the variation (63%, **Figure 2B**) in NIE as compared to the NHI (10%; **Figure 2D**). Variation in grain %N was ample, eightfold, as compared with the recorded by NHI, fivefold (variation = maximum – minimum; **Table 2**). In summary, NHI distribution was more concentrated (0.6 to 0.7, 25%–75%Q) as compared to the grain %N (12 to 19 mg g^-1^, 25–75%Q; **Table 2**); the broader range on the latter factor contributed to its dominance over the NIE process.

### Nitrogen Use Efficiency (NUE) and Grain N Sources

**FIGURE 3:**
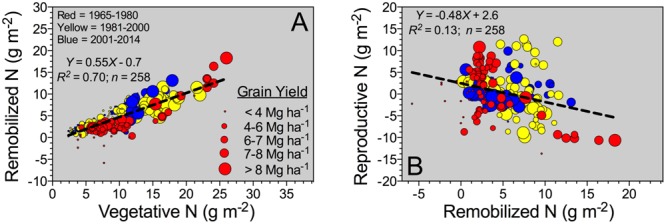
**Reproductive-stage shoot N remobilization, from silking till maturity, versus vegetative-stage whole-plant N content (Vegetative N) **(A)**, and Reproductive N versus Remobilized N **(B)** for the Old (red color: observations from 1965 to 1980; *n* = 87), Transient (yellow color: observations from 1981 to 2000; *n* = 84), and the New Eras (blue color: observations from 2001 and 2014; *n* = 87).** For both **(A,B)**, the different sizes of the bubbles correspond to grain yield values ranging from 1.2 to 20.9 Mg ha^-1^. A unified slope was fitted to represent all Eras (historical analysis).

Grain N sources were dissected to understand the physiological mechanisms underpinning NUE and its component (NIE, plant-related process). The main two mechanisms related to the grain N sources are the (herein “Remobilized N”) and the reproductive-stage aboveground plant N uptake (herein “Reproductive N”). For the pooled data, Reproductive N was close to eightfold higher than the Remobilized N (4.4 vs. 0.6 g m^-2^, respectively) (**Table 2**). Reproductive N distribution ranged from 2.2 to 6.6 g m^-2^ (25–75%Q), representing a net positive N influx (from flowering till maturity). For the counterpart, Remobilized N ranged from -1.9 to 3.0 g m^-2^ (25–75%Q), negative values were related to accumulation of stover N (N reservoir, stem fraction); while positive values portrayed a net stover N remobilization (**Table 2**). Overall plant N status attained at flowering is connected to the potential Remobilized N capacity of the plant during the reproductive period. For this study, Remobilized N was related to Vegetative N by a unique linear model, regardless of the Eras evaluated (**Figure 3A**). Therefore, Remobilized N increased as Vegetative N rose. For the Old (red color) and New (blue color) Eras, slopes of the association between Remobilized N and Vegetative N did not statistically differ (*Y* = 0.63X; *F*-test; Mead et al., 1993). For the Reproductive N and Remobilized N association, slopes for the Old and New Eras did differ statistically, with a greater trade-off (more negative slope) for the Old (*Y* = -0.75X) relative to the New (*Y* = -0.38X) Eras (*F*-test; Mead et al., 1993). The N status at flowering (Vegetative N) accounted for a large variation of the Remobilized N (*R*^2^ = 0.70); while, the Remobilized N accounted for a small part (*R*^2^ = 0.13) of the Reproductive N (**Figure 3**).

## Discussion

Yield gap from Old to New Era for the high yielding range (Δ = 0.6 Mg ha^-1^), was similar to the rate of yield increase documented by Mason et al. (2008), 0.67 Mg ha^-1^ from 1950 to 2000 years. The USDA-NASS Commodity Statistics Database (2015) documented a larger historical (1957–2000) yield gain for sorghum under irrigated conditions of 1.1 Mg ha^-1^. However, Assefa and Staggenborg (2010) documented a lack of yield increase over time (1957–2008) at irrigated sites in Kansas. As connected to yield components, Mason et al. (2008) documented a lesser contribution of seed size to sorghum yield improvement, but primarily governed by head number and grains per head. For the plant density factor, historical changes in plant density were documented in maize (Duvick and Cassman, 1999; Ciampitti and Vyn, 2012), but no changes over time were reported for sorghum crop under irrigated conditions (Assefa and Staggenborg, 2010). Notwithstanding intrarow spacing did diminish over time under dryland conditions (Assefa and Staggenborg, 2010), improvement in plant density factor was not a physiological trait related to sorghum yield gain over time (Assefa and Staggenborg, 2011).

For the plant N content to BM relationship, a proportional gain in N content was documented as plant size (BM) increases. Accordingly, Lemaire et al. (1996) reported similar relationship (and values) between plant N content and plant BM at maturity for sorghum. At a comparable biomass level, sorghum presented higher N content than maize, which seems to be intrinsically related to the species (genotypic difference) (Lemaire et al., 1996). Further research and comparative analysis is needed in order to clarify this point and clearly isolate the effect of plant size and growth/development when comparing maize versus sorghum for plant yields, N content, and NUE.

Grain yield to plant N content relationship, NIE slope, presented a unique model across historical Eras. For sorghum, grain yields ranging from 8 to 9 Mg ha^-1^, plant N content was close to 25 g m^-2^ even under diverse hybrids (short vs. tall hybrids) (van Oosterom et al., 2010). Similarly to the current synthesized grain yield-to-plant N content data, Vanderlip (1993) reported a plant N content of 20 g m^-2^ for a grain yield of 8.5 Mg ha^-1^. From the efficiency term, the NIE can be understood as the N physiological efficiency related to the utilization of N by the sorghum plant for yield production. The upper boundary line depicts maximum NIE related to N-limited environments, minimum plant N concentration and/or maximum N partitioning, herein expressed as the NHI (grain N to plant N content). On the opposite boundary, the lower line portrays minimum NIE observations related to yield-limited environments, maximum plant N concentration and/or reduced or minimum NHI (smaller bubble sizes; **Figure 2A**). The NIE variation seems to be primarily driven by changes in yield (with concomitant changes in plant N content). Similar outcome was synthesized for maize in a historical analysis (Ciampitti and Vyn, 2012, 2013), with also a unique model for all Eras evaluated. Although only a small proportion of the variation in NIE was explained from both grain HI and NHI; a similar behavior previously documented for maize (Ciampitti and Vyn, 2012).

### Nitrogen Use Efficiency (NUE) and Grain N Sources

Reproductive N presented negative values in few studies, which can be connected to larger losses via volatilization from leaf organs as compared to the net uptake (Daigger et al., 1976; Stutte et al., 1979; Farquhar et al., 1983; Harper et al., 1987; Francis et al., 1993). Variation on Remobilized N parameter is connected to greater accumulation or net remobilization from the stover organ. van Oosterom et al. (2010) reported that maximum Remobilized N from both stem and rachis (branches of head) were positively associated to their N status (organ N concentration). For maize, Remobilized N was tightly and positively connected with the N status achieved in the plant at flowering (herein “Vegetative N”) even under diverse G × E × M across a historical and global evaluation (Ciampitti and Vyn, 2013). A unique linear model fit the relationship between Remobilized N and Vegetative N across historical Eras (**Figure 3A**). The latter confirms that the association did not change in the last 60 years, but the main gain seemed to be related to a greater vegetative-growth ability connected with superior N content at the plant-level.

Increasing N remobilization is a desirable plant trait, but a clear trade-off between Remobilized N and Reproductive N was already recognized in other crops such as maize (Pan et al., 1986; Gallais and Coque, 2005; Ciampitti and Vyn, 2013) and wheat (Kichey et al., 2007; Bogard et al., 2010), and now is also documented for sorghum. Further studies are needed in order to validate that modern sorghum hybrids have a more balanced association between Reproductive N and Remobilized N (herein term “reproductive N trade-off”). Progress in diminishing the strength of the reproductive N trade-off will facilitate NUE improvement from a physiological perspective. In addition, research investigations focused on the non-reproductive organs (primarily stem and leaf) are needed for properly dissecting the role of each organ on the reproductive N trade-off mechanisms. van Oosterom et al. (2010) presented critical information on the role of each organ during the reproductive period, emphasizing the contribution of the stem and leaf organs to the Remobilized N process.

Physiological explanations on the reproductive N trade-off were previously documented and summarized for maize by Ciampitti and Vyn (2013). Briefly, a hypothesis related to the “sink-limitation”, as yield is reduced by stress factors, both Remobilized N and Reproductive N are affected via leaf senescence process, affecting the reproductive N trade-off mechanism. The latter scenario depends on the timing of the stress relative to the crop growth. If the stress occurs after flowering, Remobilized N could increase at the expenses of low or negligible Reproductive N (if grain filling duration is shortened). On the counterpart, a vegetative-stress could reduce early-season growth and N content (lower Vegetative N and Remobilized N); thus, Reproductive N could partially compensate low Vegetative N content. For maize, Pan et al. (1984) documented the importance of a balanced Remobilized N (but late-season) and Reproductive N sources. More “balanced” N sources need to be pursued for improving overall sorghum NUE from a physiological viewpoint. Changes in sorghum germplasm could modify NIE and overall NUE. Borrell and Hammer (2000) documented a proportional N allocation to the leaf organs in stay-green versus conventional (senescent) sorghum hybrids before flowering, related to a change in leaf morphology (thickness), consequently increasing leaf N demand. During the reproductive period, higher leaf N demand for stay-green hybrids was expressed as greater Reproductive N and lower Remobilized N, compensating the extra leaf N requirement as relative to senescent hybrids (Borrell and Hammer, 2000). Stay-green trait presented larger Vegetative and Reproductive N; while senescent hybrids have a larger proportion of the grain N content supplied by the Remobilized N process (rapid leaf senescence). Similar outcomes were documented for maize (Ta and Weiland, 1992; Rajcan and Tollenaar, 1999).

Reproductive N balance seems to be related to the “sink demand”, primarily driven by the number of heads and grains per head (grain m^-2^), which is partially explained from the Vegetative N and Remobilized N association (both factors increased as yield boosted, larger bubble sizes) (**Figure 3A**). van Oosterom et al. (2010) documented a fairly constant N demand right after flowering for three hybrids differing in plant height and stay-green under drought conditions. Early grain N demand (primarily sink strength driven process), even when non-dependent on the plant N supply, could potentially dictate final grain %N under optimal growing conditions. van Oosterom et al. (2010) also documented a similar early-reproductive grain N accumulation rate relative to the final grain %N, 18.2 mg g^-1^, attained at maturity. Overall, a similar grain %N was synthesized for the current historical study, 16.5 mg g^-1^ on sorghum (**Table 2**). As postulated by the van Oosterom et al. (2010), sink strength (portrayed as grains m^-2^) seems to be the primary factor governing N demand during early reproductive. Therefore, if Reproductive N cannot fulfill this grain N demand, then Remobilized N is the main process for satisfying the grain N requirement (Triboï and Triboï-Blondel, 2002). This early grain N deposition is related to structural and metabolic processes (cell division), which seems to be independent of the plant N supply level (e.g., in sorghum, van Oosterom et al., 2010; wheat, *Triticum aestivum* L., Martre et al., 2003). High Remobilized N scenarios from mid-to late-grain filling are the outcome of a grain N demand that cannot be fully sustained with the Reproductive N, which could be indirectly impacted by source-limitations (functional photosynthetic affected via leaf senescence). If the grain N demand is not fulfilled (under N deficiency) with the stem N reservoir, then leaf N remobilization could increase to overcome the stem-to-grain N gap. Similar results were previously documented in sorghum by several authors (Youngquist and Maranville, 1992; Utzurrum et al., 1998; Borrell and Hammer, 2000; van Oosterom et al., 2010). As hypothesized by van Oosterom et al. (2010), stem N remobilization is connected with its N concentration; thus, N will be translocated until its minimum N concentration is reached (so called “structural N”); from this point afterward, leaf N will become the primary source to meet the grain N demand.

From a genotypic perspective, recent studies were conducted to investigate and map QTLs under varying N levels (Gelli et al., 2016). New insights on differential expression of transcripts related to N metabolism could provide new approaches for further improving NUE. Lastly, from a biochemical perspective, a better understanding of NUE process will assist not only in yield improvement via superior nutrient efficiency but also is connected in the regulation of leaf senescence via the interplay portrayed by the C:N balance (Chen et al., 2015). Therefore, an integrated and multi-disciplinary study on NUE and its processes is needed for fully comprehend the implications on sorghum.

## Conclusion

In summary, important highlights from this synthesis are: (1) NUE component related to the plant process (NIE), was primarily explained by variations in grain %N; (2) historical changes in NUE were not directly related to physiological modifications of the yield to plant N content ratio, but primarily explained by changes in the grain %N (lower grain %N under superior yields); (3) a strong dependency exists between the Remobilized N and Vegetative N; and (4) an evident trade-off between Remobilized N and Reproductive N. These outcomes are similar to those documented for corn, suggesting similar physiological mechanisms underpinning NUE process across these two crops species with similar photosynthetic pathway (C_4_).

The reproductive N trade-off presented in this synthesis-analysis established a platform for the overall N balance sources for sorghum crop. The theory of a “path-dependence” scenario for grain N demand, N utilization, and final NUE can clearly apply when sorghum N balance is dissected in vegetative and reproductive N forces. Greater Vegetative N and more balanced N partition between the non-reproductive organs (primarily leaf vs. stem) could lessen the crop dependency on the leaf N, via utilization of the stem N reservoir, delaying leaf senescence and improving overall functional stay-green. Sustaining Reproductive N during grain filling could assist in minimizing any shortfall between grain N demand and Vegetative N supply. Continuation of N uptake is not only connected to sink-strength, but also to source-limiting factors (also impacting functional stay-green – C supply). Future research studies should focus on elucidating avenues to improve reproductive N trade-off mechanism(s), which can be potentially explored via the use of hybrids (genetics) with high-yield potential and stay-green trait.

## Author Contributions

IC collected data, designed research, performed research, analyzed, and synthesized data, and wrote the paper. PP analyzed data and wrote the paper.

## Conflict of Interest Statement

The authors declare that the research was conducted in the absence of any commercial or financial relationships that could be construed as a potential conflict of interest.

## Abbreviations

Grain %N: Grain N concentration
HI: harvest index
NHI: N harvest index
NIE: N internal efficiency
NUE: N use efficiency
Plant N: whole-plant N uptake
Remobilized N: reproductive-stage shoot N remobilization
Reproductive N: reproductive-stage whole-plant N uptake
Vegetative N: vegetative-stage whole-plant N uptake
